# Supplementary material for: Brassica napus Plants Gain Improved Salt-Stress Tolerance and Increased Storage Oil Biosynthesis by Interfering with CRL3BPM Activities
Source: Plants (Basel). 2023 Mar 1;12(5):1085. doi: 10.3390/plants12051085 (PMC10005049; doi:10.3390/plants12051085)
Supplement: Supplementary file 1 [file plants-12-01085-s001.zip › plants-2208768-supplementary.pdf]

(a)

```
AtWRI1          MKKRLTTSTCSSSPSSSVSSSTTTSSPIQSEAPRPKRAKRAKKSSPS-GDKSHNPTSPAS  59
BrWRI1.1        MKRPLTTSPSTS----SSTSSSACILPTQPETPRPKRAKRAKKSSIPTDVKPQNPTSPAS  56
BrWRI1.2        MKRPLTTCTSSST---SSSTSSSCILRNQPETPRPKRAKRAKKSSPPCDVKPQNPTSPAS  57
                **: ***. .:*    * ::*::     * *:*************   . * :*******

AtWRI1          TRRSSIYRGVTRHRWTGRFEAHLWDKSSWNSIQNKKGKQVYLGAYDSEEAAAHTYDLAAL  119
BrWRI1.1        TRRSSIYRGVTRHRWTGRYEAHLWDKSSWNSIQNKKGKQVYLGAYDSEEAAAHTYDLAAL  116
BrWRI1.2        ARRSSIYRGVTRHRWTGRFEAHLWDKSSWNSIQNKKGKQVYLGAYDSEEAAAHTYDLAAL  117
                :*****************:*****************************************

AtWRI1          KYWGPDTILNFPAETYTKELEEMQRVTKEEYLASLRRQSSGFSRGVSKYRGVARHHHNGR  179
BrWRI1.1        KYWGPDTILNFPAETYTKELEEMQRCTKEEYLASLRRQSSGFSRGVSKYRGVARHHHNGR  176
BrWRI1.2        KYWGPDTILNFPVETYTKELDEMQRGTKEEYLASLRRQSSGFSRGVSKYRGVARHHHNGR  177
                ************.*******:**** **********************************

AtWRI1          WEARIGRVFGNKYLYLGTYNTQEEAAAAYDMAAIEYRGANAVTNFDISNYIDRLKKKGVF  239
BrWRI1.1        WEARIGRVFGNKYLYLGTYNTQEEAAAAYDMAAIEYRGANAVTNFDISNYIDRLKKKGVF  236
BrWRI1.2        WEARIGRVFGNKYLYLGTYNTQEEAAEAYDMAAIEYRGANAVTNFDISNYIDRLKKKGVF  237
                ************************** *********************************

AtWRI1          PFPVNQANHQEGILVEAKQEVETREAKEEPREEVKQQYVEEPPQEEEEKEEEKAEQQEAE  299
BrWRI1.1        PFPVSQANHQEAVLAEAKQEVEA---KEEPTEEVKQCVEKEEPQEAKEEKTEKK------  287
BrWRI1.2        PFRVEQATHQEAVLAEAKQEAKE---------EVKEHVEE-EHQEAREETTEQK------  281
                ** *.**.***.:*.*****.:          ***:   :   ** .*:  *:

AtWRI1          IVGYSEEAAVVNCCIDSSTIMEMDRCGDNNELAWNFCMMDTGFSPFLTDQNLANENPIEY  359
BrWRI1.1        -----QQQQEV----EEAVVTCCIDSSESNELAWDFCMMDSGFAPFLTDSNLSSENPIEY  338
BrWRI1.2        -----QEVEAVTCGVDASGIMEMERSSDSNELAWNFCMMDSGFAPFLTDQNLSNENPIEY  336
                     ::   *    : : :     ..:.*****:*****:**:*****.**:.******

AtWRI1          PELFNEL-AFED-NIDFMFDDGKHECLNLENLDCC----VVGRESPPS-SSSPLSCLSTD  412
BrWRI1.1        PELFNEM-GFED-NIDFMFEEGKQDCLSLENLDCCDGVVVVGRESPTSLSSSPLSCLSTD  396
BrWRI1.2        PELFNEMMGFEDNDIDFMFEEAKNECLSLENLDCCDV-VVVGRESPTSLSSSPLSCFSTD  395
                ******: .*** :*****::.*::**.*******    ******* * *******:***

AtWRI1          SASSTT---TTTTSVSCNYLFQGLFVGSE        438
BrWRI1.1        SASSTTT--TTITSVSCNYSV--------        415
BrWRI1.2        SASSTTITTTTTTSVSCNYSV--------        416
                ******   ** ******* .
```

(b)

1: AtWRI1	100.00	81.37	81.28
2: BrWRI1.1	81.37	100.00	86.49
3: BrWRI1.2	81.28	86.49	100.00

Figure S1: Arabidopsis and Brassica WRI1 alignment and identity scores. (**a**) Clustal Omega based multiple amino acid sequence alignment (https://www.ebi.ac.uk/Tools/msa/ clustalo /) using standard settings. * (asterisk), indicates single, fully conserved residue; :(colon), indicates conservation between groups of strongly similar properties -scoring > 0.5 in the Gonnet PAM 250 matrix; . (period), indicates conservation between groups of weakly similar properties - scoring =< 0.5 in the Gonnet PAM 250 matrix. (**b**) Percent Identity Matrix - created by Clustal2.1

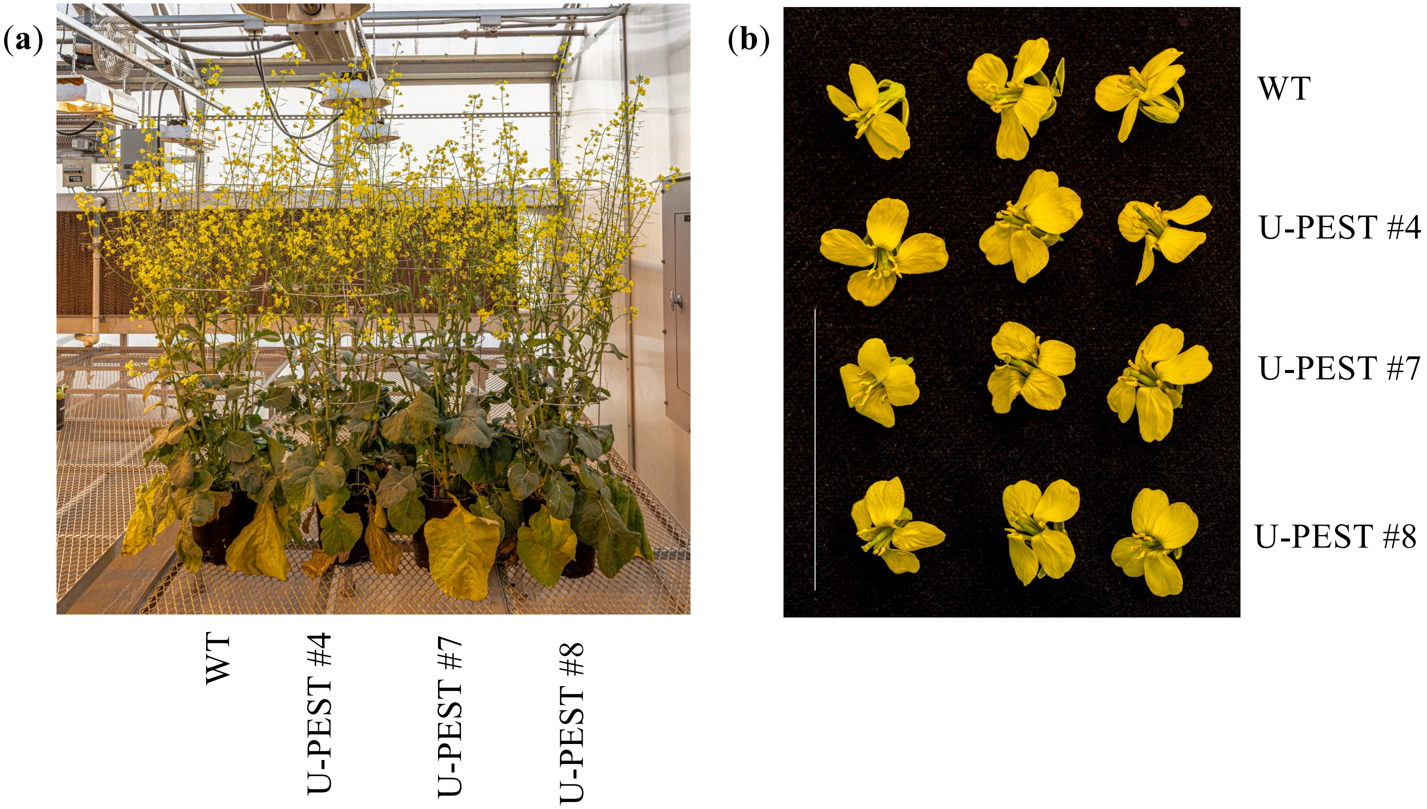

Figure S2. Phenotypes of WT and U-PEST lines. Adult, flowering WT and U-PEST plants do not show any obvious growth differences on either (**a**) the overall plant morphology or (**b**) specifically flower development. Bar = 5 cm.

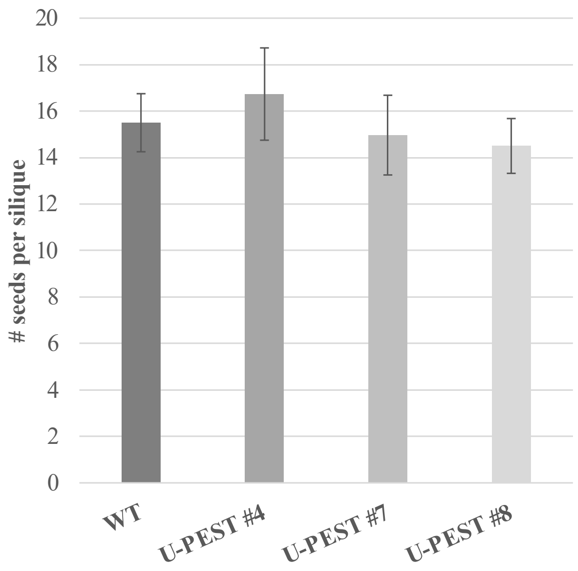

Figure S3. Seed number per silique in WT and the three U-PEST lines. No significance difference was found between the different genetic backgrounds. Data are based on three individual plants where n=10 siliques (in total 30 siliques/genetic background) were analyzed for their seed number.

Table S1: Germination data for WT and U:PEST lines under control and 150mM NaCl stress conditions. Given are the accumulated number of germinated seeds for each line on each day after plating. Final germination percentage gives the final percentage of seeds which germinated (out of 30 seeds plated). Time to 10%, 50%, and 90% Germination gives the number of days since plating for total germination to reach 10%, 50%, and 90%, respectively. Uniformity of germination gives the number of days elapsed between the first and last germination events. Mean germination time gives the mean day germination events took place. Variance of germination time gives the difference in days between the mean germination time and the last germination event. Coefficient of velocity of germination calculates how rapidly germination took place without accounting for final germination percentage. Germination rate index gives a summary value that incorporates the rate of germination and the final germination percentage. An asterisk next to the germination rate index value indicates a significant difference from WT under the same conditions (two-sample t-test, α=0.05). n=3. Calculations were done in R using the "SeedCalc" package.

**No Salt**	**Total # of Seeds Germinated**			
**Day**	**WT**	**U:PEST #4**	**U:PEST #7**	**U:PEST #8**
1	**2.4** ± 0.5	**4.3** ± 2.1	**5.7** ± 2.7	**6.1** ± 4.5
2	**15.2** ± 11.4	**10.8** ± 3.8	**12.5** ± 4.8	**21.1** ± 4.9
3	**19.8** ± 9.4	**16.9** ± 3.8	**18.5** ± 4.7	**26.1** ± 2.9
4	**26.8** ± 4.3	**23.7** ± 4.8	**24.5** ± 4.4	**27.3** ± 1.6
5	**27.8** ± 2.6	**27.0** ± 2.9	**26.2** ± 3.7	**28.4** ± 1.6
6	**29.0** ± 0.9	**28.5** ± 1.6	**27.6** ± 1.9	**28.7** ± 1.2
7	**29.0** ± 0.9	**28.5** ± 1.6	**27.6** ± 1.9	**28.7** ± 1.2
**Parameters**				
Final Germination Percentage (%)	**96.7** ± 2.9	**95.0** ± 5.4	**92.0** ± 6.2	**95.7** ± 4.0
Time to 10% Germination (days)	**1.4** ± 0.6	**< 1**	**< 1**	**< 1**
Time to 50% Germination (days)	**2.2** ± 1.0	**2.6** ± 0.6	**2.2** ± 0.6	**1.7** ± 0.2
Time to 90% Germination (days)	**3.8** ± 1.4	**4.4** ± 0.6	**4.2** ± 0.9	**3.0** ± 0.8
Uniformity of Germination (days)	**3.0** ± 0.6	**4.1** ± 0.4	**3.7** ± 0.6	**2.2** ± 1.2
Mean Germination Time (day)	**2.8** ± 0.9	**3.1** ± 0.4	**2.8** ± 0.5	**2.2** ± 0.4
Variance of Germination Time (days)	**1.0** ± 0.6	**1.9** ± 0.4	**1.9** ± 1.1	**1.0** ± 0.5
Coefficient of Velocity of Germination	**37.4** ± 10.8	**32.5** ± 4.0	**36.2** ± 6.3	**46.6** ± 8.5
**Germination Rate Index**	**12.5** ± 3.3	**12.2** ± 2.3	**13.2** ± 2.2	**15.8** ± 2.9

**150mM NaCl**	**Total # of Seeds Germinated**			
**Day**	**WT**	**U:PEST #4**	**U:PEST #7**	**U:PEST #8**
1	**0.0**	**0.5** ± 0.5	**0.9** ± 0.9	**0.9** ± 0.9
2	**0.0**	**1.1** ± 0.6	**4.1** ± 2.6	**6.4** ± 2.8
3	**0.0**	**1.6** ± 0.9	**7.7** ± 1.7	**10.8** ± 3.0
4	**0.9**	**4.5** ± 1.5	**20.4** ± 2.2	**17.8** ± 3.3
5	**0.9**	**6.1** ± 1.6	**24.3** ± 2.1	**22.5** ± 2.6
6	**1.4** ± 1.0	**6.6** ± 1.7	**25.4** ± 1.7	**23.6** ± 2.4
7	**1.4** ± 1.0	**6.6** ± 1.7	**25.4** ± 1.7	**23.6** ± 2.4
**Parameters**				
Final Germination Percentage (%)	**5.0** ± 3.5	**22.0** ± 5.6	**84.8** ± 5.7	**79.3** ± 8.3
Time to 10% Germination (days)	**3.2** ± 0.1	**1.9** ± 0.7	**1.7** ± 0.4	**1.4** ± 0.3
Time to 50% Germination (days)	**4.1** ± 1.0	**3.5** ± 0.3	**3.4** ± 0.1	**3.1** ± 0.3
Time to 90% Germination (days)	**4.6** ± 1.2	**4.9** ± 0.5	**4.7** ± 0.3	**4.7** ± 0.3
Uniformity of Germination (days)	**1.4** ± 1.0	**2.8** ± 0.6	**3.0** ± 0.5	**3.3** ± 0.3
Mean Germination Time (day)	**4.5** ± 0.8	**3.8** ± 0.4	**3.8** ± 0.2	**3.5** ± 0.3
Variance of Germination Time (days)	**1.0** ± 0.9	**2.0** ± 1.1	**1.2** ±0.4	**1.6** ± 0.4
Coefficient of Velocity of Germination	**22.5** ± 3.5	**26.3** ± 2.6	**26.7** ± 1.5	**28.5** ± 2.7
**Germination Rate Index**	**0.3** ± 0.2	**2.1** ± 0.4*	**7.8** ± 1.3*	**8.0** ± 1.5*

Table S2: List of primers used in the study

Cloning	
Name	sequence
**UBQFWattb1**	AAAAAGCAGGCTTAATGCAGATCTTCGTCAAG
**BrRAP2.4-1attb2**	AGAAAGCTGGGTTCAAGGCAAAATCGAA
**proBrWRI1.2 FW**	CAGAAATATATATGCTGCCA
**proBrWRI1.2*Hind*IIIFWL**	GGCAGGCTTTAAGAAGGAGATATACATATGAAGCTTCAGAAATAT ATATGCTGCCA
**proBrWRI1.2*Kpn*IRW**	ATGATATGTATATCTCCTTCTTAAGGTACCTAAATTCTGAGAAAAA AGCT
**proBrWRI1.2 RW**	TAAATTCTGAGAAAAAAGCT
**proBraWRI1.2attb1**	AAAAAGCAGGCTATAAGCTTACAGAAATATATATGCTGCCA
**proBraWRI1.2attb2**	AGAAAGCTGGGTTAAATTCTGAG
RT-qPCR Analysis	
**qRTWRI1.2FW**	GGATAACGACATAGACTTCAT
**qRTWRI1RW**	ACAACGACATCACAACAA
**GUbPESTqFWUb**	GTTGAGTCTTCAGACACCATCG
**GUbPESTqRW205**	ACAGCCAAGCTCCAGATCCG
**ActinAF111812RTqPCRFW**	TGGGTTTGCTGGTGACGAT
**ActinAF111812RTqPCRRW**	TGCCTAGGACGACCAACAATACT
